# Impact of Establishing a Cardiology Fellowship at a Rural Academic Medical Center

**DOI:** 10.1016/j.jacadv.2026.103044

**Published:** 2026-07-20

**Authors:** Apurva Popat, Brian Finnegan, Deepa Soodi, Humberto Vidaillet, Param P. Sharma

**Affiliations:** a Department of Cardiology, Sanford Health, Marshfield Clinic, Marshfield, Wisconsin, USA; b Marshfield Clinic Research Institute, Sanford Health, Marshfield Clinic, Marshfield, Wisconsin, USA

**Keywords:** cardiology fellowship, graduate medical education, rural health

Cardiovascular disease remains the leading cause of death in the United States, but access to cardiovascular care is not distributed evenly. Rural communities continue to face longer travel distances, fewer specialists, and greater barriers to timely diagnostic and therapeutic care. Recent national analyses show that nearly half of U.S. counties have no practicing cardiologist, and more than 86% of rural counties are without one. Counties lacking cardiologists have worse cardiovascular mortality, emphasizing that workforce distribution is not only a staffing issue but also a possible patient-outcome issue.^1^, ^2^, ^3^

That national picture is highly relevant in central Wisconsin, where Marshfield Medical Center serves as a major referral hub for a largely rural region. Marshfield is a city of about 18,973 people, yet Marshfield Medical Center functions far beyond the scale of its home city. The surrounding area is rooted in small-town communities, with family-owned dairy farming remaining one of the region’s primary industries. It is a 298-bed hospital, the only hospital within a 29-mile radius, and serves Wood County and surrounding communities. Wood County itself has 73,939 residents, and more than one-third of those residents live in rural areas. The broader Marshfield Clinic region extends across Wisconsin and Michigan’s Upper Peninsula through a network of more than 60 locations, with approximately 3.2 million patient encounters annually and 310,000 unique patients.^4^

The purpose of this article is to describe the rationale, development, and early institutional impact of establishing a cardiovascular disease fellowship within a rural health system, and to explore how graduate medical education may serve as a strategy to preserve specialty care access in underserved communities.

In this setting, the decision to build a cardiovascular disease fellowship was driven by 3 practical needs: sustaining cardiovascular services, improving patient access, and preserving an academic mission that could support long-term recruitment and retention. Rural systems often experience prolonged vacancies, growing call burden, increasing reliance on locums, and difficulty recruiting subspecialists trained in large urban programs. A fellowship cannot solve each of these problems immediately. However, it can create a local training pipeline, expand clinical capacity, and strengthen the academic environment that makes recruitment more realistic.

Our institutional data suggest that the fellowship became an important capacity-preserving strategy during a period of declining attending workforce. Although cardiology attending outpatient clinical full-time equivalent (FTE) fell substantially from 23.8 in 2020 to 2021 to 12.5 in 2024 to 2025, overall outpatient visit volume remained relatively stable, with 13,439 visits in 2020 to 2021 and 12,130 visits in 2024 to 2025. By 2024 to 2025, fellows contributed directly to this effort, accounting for 1,154 outpatient appointments and 862 unique patients. Using 8 clinical hours per FTE, this corresponds to a reduction from 190.4 to 100.0 attending clinical hours, representing a loss of 90.4 hours of attending clinical capacity (47.5%). In the absence of fellowship support, a reduction of this magnitude would reasonably be expected to result in lower outpatient capacity, fewer available appointments, longer wait times, reduced continuity, and diminished access for rural patients. Taken together, these findings suggest that fellowship integration helped the program continue meeting patient care needs despite a shrinking cardiology workforce.

The same pattern was seen in the acute setting. Acute authored documents (history and physical examination notes, inpatient consult notes, progress notes, and cardiology procedure or operative notes) rose from 11,104 in 2021 to 2022 to 19,055 in 2024 to 2025, while cardiology attending clinical FTE fell from 28.075 to 15.6. Fellow-authored acute documents reached 4,037 in 2024 to 2025. Hospital consult volume also rose from 1,818 to 2,603 over the same period, again despite declining attending clinical FTE, with fellow consults reaching 1,827 in 2024 to 2025. Although before-and-after observational data cannot prove causality, these trends strongly suggest that the supervised fellowship helped preserve service capacity during a time of physician attrition.

Importantly, expansion of trainee involvement was not associated with any apparent deterioration in broad hospital metrics. Average length of stay remained stable, from 4.33 days in 2018 to 2019 to 4.10 days in 2024 to 2025. The readmission rate was 15.4% before fellowship implementation and 13.9% in 2024 to 2025, with the case-mix index remaining broadly similar. Outpatient patient-experience scores remained favorable overall. Fellowship-associated clinics achieved mean patient-experience scores of 4.85 (14 responses) in 2023 to 2024 and 4.53 (99 responses) in 2024 to 2025 on a 5-point scale, compared with nonfellowship clinic scores ranging from 4.70 to 4.73. While these are not definitive quality outcomes, they are reassuring and argue against the notion that a new fellowship necessarily disrupts care.

The benefits of a rural fellowship are not limited to service volume. Rural programs can offer broader clinical exposure, earlier procedural responsibility, close faculty mentorship, and a direct connection between trainee effort and community impact. In our experience, a cardiology fellowship enhances exposure for internal medicine residents, expands research participation, supports advanced electives and focused boot camps, and improves the visibility of cardiovascular teaching within the institution. Fellows also viewed the smaller rural training environment as a strength, noting that close faculty attention, robust hands on experience, direct mentorship, and individualized teaching created a highly personalized educational experience.

At the same time, a central concern in using fellowship training as a workforce strategy is whether educational quality and supervision can be preserved when clinical attending FTE declines. At our institution, the decline in clinical FTE did not translate into absence of faculty depth or loss of educational oversight. The cardiology division continued to maintain sufficient qualified faculty to meet Accreditation Council for Graduate Medical Education cardiovascular disease fellowship requirements, including the required program leadership, core faculty structure, supervision, teaching, evaluation, and mentorship infrastructure.^5^ This distinction is important when comparing fellows with advanced practice providers (APPs). APPs are essential members of rural cardiovascular teams and should be viewed as complementary rather than competing workforce solutions. However, an APP-only expansion does not create a future cardiologist workforce, does not fulfill the same procedural and diagnostic training mission, and may be difficult to scale in rural specialty markets where APP recruitment is also highly competitive. In addition, Medicare may help offset some of the cost of training fellows through graduate medical education payments. Because cardiology fellows have already completed their initial residency training, they are generally counted as 0.5 FTE for direct graduate medical education payment purposes. Separately, rural track program rules may allow eligible hospitals to receive support for training time in rural settings when Medicare requirements are met.^6^

That said, building such a program requires a great amount of time, institutional commitment, and coordinated effort. The barriers are substantial and should not be dismissed. Accreditation requirements are rigorous. Programs must demonstrate adequate faculty depth, case volume, administrative support, protected educational time, scholarly infrastructure, and a curriculum mapped to Accreditation Council for Graduate Medical Education milestones. Financial investment is real, and so is the cultural work required to align clinical infrastructure with educational goals. Faculty engagement, mentorship systems, and institutional buy-in are not optional; they are foundational.

The larger lesson is critically important here. In rural cardiovascular care, training capacity should be viewed as part of the care-delivery model itself. When thoughtfully designed, a fellowship can expand access, stabilize local services, improve recruitment prospects, and preserve academic identity in communities that otherwise risk progressive subspecialty loss. For rural health systems facing retiring cardiologists, difficulty recruiting, and rising cardiovascular demand, the question may no longer be whether a fellowship is an academic luxury.^7^ It may be whether they can afford not to build one.

## Funding support and author disclosures

The authors have reported that they have no relationships relevant to the contents of this paper to disclose.
